# Ophthalmic Surgery in Prion Diseases

**DOI:** 10.3201/eid1301.061004

**Published:** 2007-01

**Authors:** Tsuyoshi Hamaguchi, Moeko Noguchi-Shinohara, Yosikazu Nakamura, Takeshi Sato, Tetsuyuki Kitamoto, Hidehiro Mizusawa, Masahito Yamada

**Affiliations:** * Kanazawa University Graduate School of Medical S cience, Kanazawa, Japan; † Jichi Medical University, Shimotsuke, Japan; ‡ National Center for Neurology and Psychiatry, Ichikawa, Japan; § Tohoku University Graduate School of Medicine, Sendai, Japan; ¶ Tokyo Medical and Dental University, Tokyo, Japan; 1 Present affiliation: Ishikawa Prefecture Central Hospital, Kanazawa, Japan.

**Keywords:** Prion diseases, prion, Creutzfeldt-Jakob disease, ophthalmic surgery, cataract, dispatch

## Abstract

Eleven (1.8%) of 597 patients underwent ophthalmic surgery within 1 month before the onset of prion disease or after the onset. All ophthalmologists reused surgical instruments that had been incompletely sterilized to eliminate infectious prion protein. Ophthalmologists should be aware of prion diseases as a possible cause of visual symptoms and use disposable instruments whenever possible.

Visual impairment occurs in 10% to 20% of patients with sporadic Creutzfeldt-Jakob disease (sCJD) during an early stage of the disease (Heidenhain variant) (*1*,*2*). Some patients with prion diseases may visit ophthalmologists with visual impairment due to prion diseases or with coexisting age-related eye diseases (*3*,*4*).

Infectious prion protein (PrP^Sc^) was identified in the retina and optic nerve in patients with variant CJD (vCJD) and sCJD (*5*,*6*), and CJD has been transmitted by corneal transplantation (*7*,*8*). In the World Health Organization (WHO) guidelines, eyes were classified as highly infectious tissues (*9*).

Secondary transmission of PrP^Sc^ through ophthalmic surgery could possibly be prevented around the onset of prion diseases, although surgery that is performed long before the onset of prion diseases would not have that potential. It is important to understand the current status of ophthalmic surgery for patients with prion diseases and to clarify the clinical features of the patients with prion diseases who undergo ophthalmic surgery. Here, we describe the relevant data from CJD surveillance in Japan.

## The Study

We analyzed the patients with prion diseases who had been registered by the CJD Surveillance Committee in Japan from April 1999 through March 2005. We prospectively investigated each patient with a surveillance protocol that assembled information about life history, previous medical history, clinical history, laboratory data, and results of molecular genetic and pathologic analyses. Written consent, approved by the Institutional Ethics Committee, was obtained from all the patients’ families; members of the Surveillance Committee examined the patients and collected the data.

We classified the patients into 4 categories: sCJD, infectious prion diseases, inherited prion diseases, and unclassified prion diseases. sCJD was diagnosed according to the classical criteria established by Masters et al. (*10*). Infectious prion diseases included CJD associated with cadaveric dura mater graft (dCJD) or other iatrogenic opportunities for prion infection, in which the criteria for sCJD were applied for the diagnosis, and vCJD, in which the diagnosis was based on WHO criteria (2001) (*11*). Regarding the accuracy of the diagnosis of inherited prion diseases, cases verified by pathology report were defined as definite, and cases with mutations in the prion protein gene and neuropsychiatric manifestations compatible with prion diseases were defined as probable.

Among patients with a history of ophthalmic surgery, we directed special attention to the patients who had a history of eye surgery within 1 month before the obvious onset of prion disease or after the onset. Because the onset of prion diseases often overlaps with various kinds of prodromal symptoms, determining the precise time point of onset is difficult; therefore, we included the period of 1 month before the obvious onset. To gather information about the ophthalmic surgery, we mailed questionnaires to the ophthalmologists who operated on these patients, requesting the following information: diagnosis of ophthalmologic diseases, surgical procedures performed, changes in the symptoms after the surgery, whether the instruments were reused, and methods of cleaning reused instruments.

To ascertain the clinical features of prion diseases, we analyzed the patient’s age at onset and duration of disease course, which was calculated as the interval between the onset and the appearance of the akinetic mutism state or death in the patients who died without akinetic mutism. Among early clinical manifestations of prion diseases, dementia and visual disturbance are major determinants that would influence the indication for ophthalmic surgery, so we grouped the patients according to whether they had sought treatment for dementia or visual impairment within 2 months after onset of symptoms.

The sex distribution of the patients who had ophthalmic surgery around the time of onset of clinical symptoms and those who did not was compared by Fisher exact tests, and differences in age at onset and disease duration were compared by Mann-Whitney U tests. We used χ^2^ tests to compare the distribution of the patients with or without dementia or visual impairment within 2 months of onset. Statistical significance was defined as p < 0.05.

We found 597 patients with definite or probable diagnosis of prion diseases: 468 (78.4%) with sCJD; 78 (13.1%) with inherited prion diseases; 48 (8.0%) with infectious prion diseases, including 47 cases of dCJD; and 1 patient with vCJD and 3 patients with unclassified CJD.

Thirty-seven patients (6.2%) had a history of ophthalmic surgery at some time in their lives. Among them, 11 patients (1.8%) underwent ophthalmic surgery within 1 month before the obvious onset of prion disease or after the onset. Except for 1 patient with Gerstmann-Sträussler-Scheinker disease, all of these patients had sCJD. There have been no reports of the development of prion diseases in patients who underwent ophthalmic surgery after the ophthalmic surgery of patients with prion diseases.

Ten patients with sCJD underwent ophthalmic surgery within 14 months of symptom onset, and 8 of them had onset within 4 months of surgery (Table 1). At clinical onset, 4 patients exhibited visual symptoms, 5 had dementia, and 1 patient had a gait disturbance. All patients underwent surgery for cataracts, except for 1 patient who underwent surgery for a detached retina. According to the reports on the surgical outcome by the ophthalmologists of 7 patients, visual disturbance was unchanged in 2 patients, deteriorated in 1, and improved to some extent in 4 after surgery. All ophthalmologists reused some surgical instruments and cleaned instruments by either autoclaving or the ethylene oxide gas method, which have been reported to incompletely sterilize PrP^Sc^ (*9*,*12*).

**Table 1:** Characteristics of sCJD patients and ophthalmic surgery*

Patient no.	Sex/age, y†	Disease duration, mo‡	Symptom at sCJD onset	Ophthalmic disease	Interval, mo§	Visual symptoms after surgery	Reused instruments	Cleaning method
1	M/81	8	Visual	Cataract	4	NA	NA	NA
2	M/61	15	Dementia	Cataract	0	Improved	Yes	Autoclave (135°C for 9 min)
3	F/64	20	Visual	Cataract	14	Not changed	Yes	EOG
4	F/59	3	Dementia	Detached retina	–1	Improved	Yes	EOG
5	F/57	10	Dementia	Cataract	10	NA	NA	NA
6	F/79	5	Dementia	Cataract	–1	Improved	Yes	EOG
7	M/74	16	Visual	Cataract	3	Improved	Yes	Autoclave (132°C for 10 min), EOG
8	F/63	5	Visual	Cataract	1	Deteriorated	Yes	Autoclave (132°C for 10 min)
9	M/79	6	Gait disturbance	Cataract	2	Not changed	Yes	Autoclave (121°C for 60 min)
10	F/66	3	Dementia	Cataract	1	NA	NA	NA

*sCJD, sporadic Creutzfeldt-Jakob disease; visual, visual impairment; NA, not available; EOG, ethylene oxide gas. †At sCJD onset. ‡Disease duration, the duration from onset to akinetic mutism state or death if the patients never displayed akinetic mutism. §Between surgery and sCJD symptoms.

Clinical features were compared between sCJD patients who did and did not have ophthalmic surgery (Table 2). The patients who had ophthalmic surgery had a significantly longer disease duration than those without (p = 0.0004). Among those who had early clinical symptoms within 2 months after onset, the subgroup with visual symptoms without dementia who had ophthalmic surgery was significantly more likely to have had early symptoms than those who did not have surgery (p = 0.0004).

**Table 2:** Clinical symptoms of sCJD within 2 mo after disease onset*

Characteristic	Ophthalmic surgery		Total	p value
	No, n = 458	Yes, n = 10		
Female/male	263/195	6/4	269/199	0.57
Age at onset, y; mean ± SD	66.8 ± 9.9	68.3 ± 9.1	66.8 ± 9.9	0.74
Disease duration,† mean ± SD	4.2 ± 4.8	9.1 ± 6.0	4.3 ± 4.9	0.0004
Clinical symptoms (%)				
Dementia (+)/visual impairment (+)	153 (34.2)	4 (40.0)	157 (34.3)	
Dementia (+)/visual impairment (–)	239 (53.3)	3 (30.0)	242 (52.8)	0.0004
Dementia (–)/visual impairment (+)	16 (3.6)	3 (30.0)	19 (4.1)	
Dementia (–)/visual impairment (–)	40 (8.9)	0	40 (8.7)	

*sCJD, sporadic Creutzfeldt-Jakob disease; SD, standard deviation; +, with; –, without. †Disease duration, the duration from onset to akinetic mutism or death if patients never displayed akinetic mutism.

## Conclusion

Our study showed that, in 1.8% of the patients with prion diseases, eye tissues were operated on within 1 month before the obvious onset of prion disease or after the onset. In addition, the sCJD patients who underwent surgery had a significantly longer duration of the disease course as well as significant overrepresentation of visual symptoms without dementia in the early phase, compared with patients who did not have ophthalmic surgery.

The prevalence of ophthalmic surgery around the time of clinical onset of prion diseases in our study is similar to that (2.0%) in a report from the United Kingdom (*13*). In the UK study (*13*), patients with Heidenhain variant cases constituted 40% of sCJD patients who had ophthalmic surgery. Early visual impairment (due to prion diseases) would prompt ophthalmologists to perform surgery.

Currently, cataract surgery is recommended to improve physical or cognitive function in elderly patients (*14*,*15*). It should be noted that, after performing eye surgery on patients with prion disease, all ophthalmologists reused surgical instruments that were sterilized with procedures that are incomplete for the sterilization of PrP^Sc^, although the WHO infection control guidelines for prion diseases (*9*) strongly recommend single-use surgical instruments for procedures involving highly infective tissues. The fact that no secondary iatrogenic cases that could be attributed to surgical procedures were found during our investigation does not diminish the need for ophthalmologists to be aware of CJD as a cause of visual symptoms (including symptoms mimicking those of cataracts) and highlight the importance of using disposable instruments whenever possible to avoid cross-contamination.
